# Economic burden made celiac disease an expensive and challenging condition for Iranian patients

**Published:** 2017

**Authors:** Mohamad Amin Pourhoseingholi, Mohammad Rostami-Nejad, Farnoush Barzegar, Kamran Rostami, Umberto Volta, Amir Sadeghi, Zahra Honarkar, Niloofar Salehi, Hamid Asadzadeh-Aghdaei, Ahmad Reza Baghestani, Mohammad Reza Zali

**Affiliations:** 1 *Gastroenterology and Liver Diseases Research Center, Research Institute for Gastroenterology and Liver Diseases, Shahid Beheshti University of Medical Sciences, Tehran, Iran*; 2 *Basic and Molecular Epidemiology of Gastrointestinal Disorders Research Center, Research Institute for Gastroenterology and Liver Diseases, Shahid Beheshti University of Medical Sciences, Tehran, Iran*; 3 *Student Research Committee, Gastroenterology and Liver Diseases Research Center, Research Institute for Gastroenterology and Liver Diseases, Shahid Beheshti University of Medical Sciences, Tehran, Iran*; 4 *Department of Gastroenterology, Milton University Hospital, UK*; 5 *Department of Medical and Surgical Sciences, Diagnostic and experimental, University of Bologna, Italy*; 6 *Gastroenterology Department, Modarres Hospital, Shahid Beheshti University of Medical Sciences, Tehran, Iran*; 7 *Gastroenterology Department, Atiyeh Hospital, Tehran, Iran*; 8 *Department of Biostatistics, Shahid Beheshti University of Medical Sciences, Tehran, Iran*

**Keywords:** Celiac, Burden, Medical cost, Iran

## Abstract

**Aim:**

The aim of this study was to estimate the economic burden of celiac disease (CD) in Iran.

**Background:**

The assessment of burden of CD has become an important primary or secondary outcome measure in clinical and epidemiologic studies.

**Methods:**

Information regarding medical costs and gluten free diet (GFD) costs were gathered using questionnaire and checklists offered to the selected patients with CD. The data included the direct medical cost (including Doctor Visit, hospitalization, clinical test examinations, endoscopies, etc.), GFD cost and loss productivity cost (as the indirect cost) for CD patient were estimated. The factors used for cost estimation included frequency of health resource utilization and gluten free diet basket. Purchasing Power Parity Dollar (PPP$) was used in order to make inter-country comparisons.

**Results:**

Total of 213 celiac patients entered to this study. The mean (standard deviation) of total cost per patient per year was 3377 (1853) PPP$. This total cost including direct medical cost, GFD costs and loss productivity cost per patients per year. Also the mean and standard deviation of medical cost and GFD cost were 195 (128) PPP$ and 932 (734) PPP$ respectively. The total costs of CD were significantly higher for male. Also GFD cost and total cost were higher for unmarried patients.

**Conclusion:**

In conclusion, our estimation of CD economic burden is indicating that CD patients face substantial expense that might not be affordable for a good number of these patients. The estimated economic burden may put these patients at high risk for dietary neglect resulting in increasing the risk of long term complications.

## Introduction

 Celiac disease (CD), also known as gluten-sensitive enteropathy, is a chronic autoimmune disorder of the small intestine caused by reaction to gluten. Gluten is a protein that is commonly found in wheat, rye and barley. Exposure to gluten leads to an inflammatory response and causes the production of several autoantibodies that can damage the small bowel mucosa and other organs.

CD is now affecting up to 1% of the population. It has been predicted that the number of CD patients, among Mediterranean, in 2020 will be 5 million, indicating an 11% increase in comparison with 2010 (1-3). 

Although atypical CD is now better recognized than decades ago, still it may take a long time and cause the patients to undergo many expensive medical investigations before an accurate diagnosis (4). On average, it takes 4 to 13 years to diagnose CD; therefore, the burden caused by undiagnosed patients is remarkable (5). The only therapy for CD is a gluten free diet (GFD). The rise in CD incidence led to the increase in the demand for GFD food. On a gluten-free diet, consumption of painkillers, antibiotics and medications for dyspepsia will decrease (5). There have been many studies showing that there is a good availability of GFD foods, but they are signiﬁcantly more expensive than non-GFD alternatives (6, 7). 

CD diagnosis also may incur medication and hospitalization costs. Although the medical costs including diagnosis and treatment of CD is high, the overall health status of these patients is excellent in comparison to other chronic medical conditions (8). In a retrospective study in the USA, the estimated direct cost of CD (included the outpatient costs and hospitalization) was $12,217 per year (9).

To find out the economic burden of CD in Iran, we have performed a cost survey to estimate direct medical costs (including hospitalization, medication and diagnostic tests) and cost of gluten free foods. 

## Methods

**Targeted population**

This was a cross-sectional study on Iranian adult celiac patients. The sample population was all adults (18 years or older) CD patients from three different cities (Tehran, Ilam and Shahrekord) who registered in Celiac disease department, Research Institute for Gastroenterology and Liver Diseases, Shahid Beheshti University of Medical Sciences, Tehran, Iran. The selected patients were interviewed using a questionnaire including demographic information and questions regarding economic burden of CD in year 2017. Each participant provided an informed consent for participation in the study and those who did not accept to participate in the interview, excluded from the study. Also incomplete questionnaires were omitted from the analysis. 

Ethic committee of Research Institute for Gastroenterology and Liver Diseases provided approval for this study. 

**Cost analysis**

In this cross-sectional study, the direct medical cost and GFD cost of Celiac were estimated. The factors used for cost estimation included frequency of health resource utilization and gluten free diet basket. 

Health resource utilization included number of physician visits in a year, medical and laboratory tests (pathology, blood test, genetic test, radiology, endoscopy and colonoscopy), hospitalization and drugs. 

The unit cost of different health resources including physician visits, laboratory tests, medication fees and the costs of hospitalization per night in one year were calculated based on the price lists approved in 2016-2017 by the Iranian Cabinet for the Public and Private Health Centers (10). 

The price of drugs was retrieved from the drug list of Food and Drug Office of Iranian Ministry of Health and Medical Education in same year (11). 

To calculate the cost of GFD, we collected all potential gluten free production and also the other foods which could be included in the food basket of a CD patient. To evaluate this check list, in a focus group (one statistician, one epidemiologist, one immunologist and two gastroenterologists) the checklist was under debate and discussion. Finally, the basket included; rice flour, corn flour, bread flour, cake flour, macaroni, biscuit, soup, meat, fish, chicken, cake, wafer, Toast, toothpaste, chocolate, tomato paste, fruits, vegetable, potato and oil. The minimum prices for each product (with the selected unit of Kg or package) were determined according to the average prices of Iranian market for year 2016-2017. Total costs were the sum of all categories (medical cost, GFD cost and loss productivity). This methodology was the same as cost analysis for functional bowel disorders (12), non-alcoholic Fatty liver disease (13) and chronic Hepatitis C (14) which have been done for Iranian patients. 

Purchasing Power Parity Dollar (PPP$) was used in order to make inter-country comparisons. PPP$ is an economic technique used when attempting to determine the relative values of two currencies. It is useful because the amount of goods, which a currency can purchase within two nations often varies drastically; it depends on availability of goods, demand for the goods, and a number of other difficult to determine factors. According to the reports released by the Iranian Central Bank and World Bank Organization in 2016-2017 (15); One PPP$ was estimated around 15234 Rials in 2017. This was then used to convert costs from Iranian Rials to PPP$. Using PPP$ is preferred to the US $, based on usual exchange rates, and make cross-country comparison of the costs more reliable.

**Statistical Analysis**

Descriptive statistics and frequency distribution including mean, standard deviation and percentage were employed. T-test and Analysis of Variance were used to test the differences between means of CD costs across patients’ demographics. P < 0.05 was considered as statistically significant. All analysis carried out using SPSS software. 

## Results

A total number of 213 celiac patients entered to this study. The mean age (± standard deviation) of patients was 29.01±15.24 and the mean age (± standard deviation) at the time of CD diagnosis was 17.15±15.75 years, respectively. 72.5% of patients were female, 52.1% were married, most of them had under high school education, and most of the patients had access to the medical insurance (Table 1). 

The mean (standard deviation) of total cost per patient per year was 3377 (1853) PPP$. This total cost including direct medical cost (medical appointments, hospitalization, investigations like endoscopies, etc.). The mean and standard deviation of medical and GFD expenses were estimated around 195 (128) PPP$ and 932 (734) PPP$, respectively. 

Table 2, is presenting the CD’s expenses across demographic factors of CD patients. According to the analysis, the total costs of CD was significantly higher for male. Also GFD cost and total cost were higher for unmarried patients. The other demographic factors were not associated with extra expenses.

## Discussion

In this study we attempted to estimate the cost of medical care and GFD per CD patient per year. Very limited studies are available in the literature to compare with these observation in Iran and to the best of our knowledge, there is no report on the economic burden of CD in non-European countries. Our study suggests a significant increase in life expenses for CD patients that include investigations and GFD costs. An American study estimated outpatient costs; 1,457$ and total costs 3,964$ per year respectively, which is higher than Iranian estimated report. This would be due to the different price of medical facilities in United States which is a higher income country compared to Iran (16). 

For some reasons the total costs were higher in men. This result however is consistent with the report of Long et al, who also found a significant higher economic burden among males with CD (7). There is not an obvious explanation for this finding. Sex-related differences in both clinical presentations of CD and comorbidities may affect the cost and expenses of care (18). 

**Figure 1:**
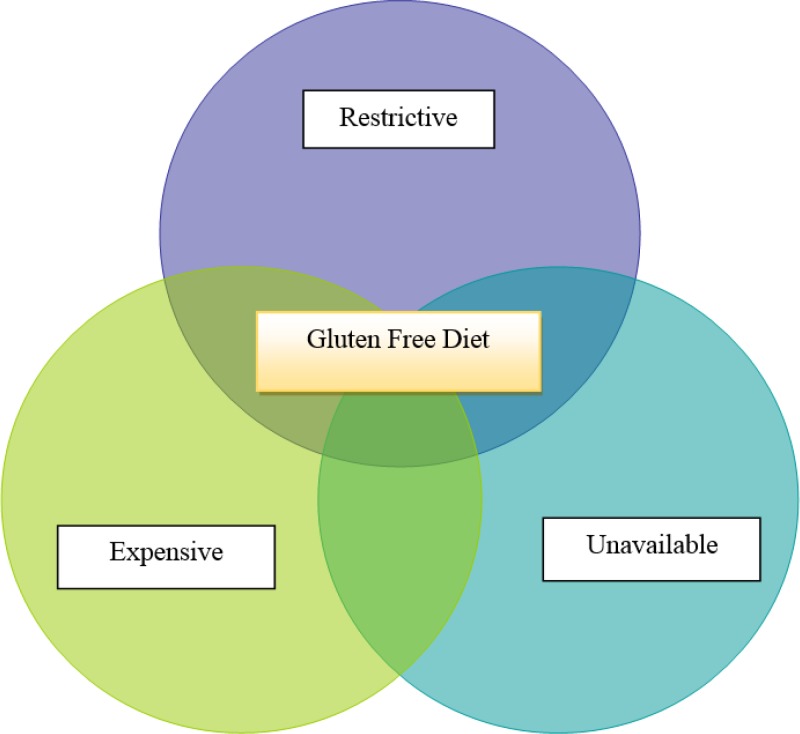
Gluten free diet Challenges

**Table 1:** Descriptive characteristics of registered celiac patients who entered to the study

Variables		Frequency	Percent
Sex	Male	58	27.5
	Female	153	72.5
Age	<30 years old	111	52.1
	>30 years old	102	47.9
Marriage	Married	111	52.1
	Not married	102	47.9
Education	Illiterate	25	12
	Under high-school	61	29.3
	Above high-school	43	20.7
	Academic	79	38
Insurance	Yes	170	79.8
	No	43	20.2

**Table 2:** The mean (SD) of CD costs according to demographic of patients (Costs were Expressed in PPP$)

Variables		GFD cost	P.Value	Medical Cost	P.Value	Total cost	P.Value
Sex	Male	226 (157)	0.073	1095 (1202)	0.17	3941 (2335)	0.022
	Female	184 (114)		870 (463)		3170 (1610)	
Age	<30 years old	205 (127)	0.271	975 (878)	0.38	3484 (1845)	0.383
	>30 years old	185 (129)		889 (561)		3260 (1864)	
Marriage	Married	178 (113)	0.034	862 (330)	0.17	3086 (1435)	0.021
	Not married	215 (140)		1008 (1014)		3693 (2185)	
Education	Illiterate	177 (99)	0.892	839 (88)	0.76	3024 (1171)	0.813
	Under high-school	194 (144)		1010 (1111)		3372 (2206)	
	Above high-school	193 (130)		943 (855)		3459 (2149)	
	Academic	201 (124)		901 (381)		3399 (1533)	
Insurance	Yes	196 (127)	0.934	955 (828)	0.37	3409 (1907)	0.612
	No	194 (132)		842 (141)		3248 (1637)	

In addition, there was not a significant difference of costs between the two age groups (under and above 30 years old). But our findings suggested that the marriage status has a considerable effect on GFD costs and total costs with significantly higher burden in single patients. These results are in agreement with the findings of Blumberg et al (2014) who expressed that married men are more likely to seek preventive health care advise because of their spouse’s encouragements (10). In addition, it was found that education and access to medical insurance had no association with economic burden of CD in Iranian patients. 

The cost of living for patients on gluten free products is much higher and it is quite challenging for people with lower income. The products available to European countries on prescription are not available to the majority of Iranian patients (Figure 1). From a patient’s perspective the availability of GF products does not always square with accessibility, i.e. being at the shop shelf at the point of need. The government’s support with prescription of GF products will be required to ensure a safer transition to GFD for newly diagnosed CD patients and preventing the potential long-term complications that may results from dietary neglect.

## Conflict of interests

The authors declare that they have no conflict of interest.

## References

[1] Greco L, Timpone L, Abkari A, Abu-Zekry M, Attard T, Bouguerrà F (2011). Burden of celiac disease in the Mediterranean area. World J Gastroenterol.

[2] Rostami Nejad M, Rostami K, Pourhoseingholi MA, Nazemalhosseini Mojarad E, Habibi M, Dabiri H (2009). Atypical presentation is dominant and typical for coeliac disease. J Gastrointestin Liver Dis.

[3] Rostami K, Bold J, Parr A, Johnson MW (2017). Gluten-Free Diet Indications, Safety, Quality, Labels, and Challenges. Nutrients.

[4] Green PHR, Stavropoulos SN, Panagi SG, Goldstein SL, McMahon DJ, Absan H (2001). Characteristics of adult celiac disease in the USA: results of a national survey. Am J Gastroenterol.

[5] Ukkola A, Kurppa K, Collin P, Huhtala H, Forma L, Kekkonen L (2012). Use of health care services and pharmaceutical agents in coeliac disease: a prospective nationwide study. BMC Gastroenterol.

[6] Burden M, Mooney PD, Blanshard RJ, White WL, Cambray-Deakin DR, Sanders DS (2015). Cost and availability of gluten-free food in the UK: in store and online. Postgrad Med J.

[7] Lee AR, Ng DL, Zivin J, Green PH (2007). Economic burden of a gluten-free diet. J Hum Nutr Diet.

[8] Shah S, Akbari M, Vanga R, Kelly CP, Hansen J, Theethira T (2014). Patient perception of treatment burden is high in celiac disease compared with other common conditions. Am J Gastroenterol.

[9] Guandalini S, Tundia N, Thakkar R, Macaulay D, Essenmacher K, Fuldeore M (2016). Direct costs in patients with celiac disease in the USA: A retrospective claims analysis. Dig Dis Sci.

[10] Medical Lows and Tariffs (2016-2017). The price list by Iranian cabinet of the public health centers.

[11] Iranian Ministry of Health and Medical Education (2016-2017). List of price of drugs.

[12] Moghimi-Dehkordi B, Vahedi M, Pourhoseingholi MA, Khoshkrood Mansoori B, Safaee A, Habibi M (201). Economic burden attributable to functional bowel disorders in Iran: a cross-sectional population-based study. J Dig Dis.

[13] Ghamar Chehreh ME, Vahedi M, Pourhoseingholi MA, Ashtari S, Khedmat H, Amin M (2013). Estimation of diagnosis and treatment costs of non-alcoholic Fatty liver disease: a two-year observation. Hepat Mon.

[14] Ashtari S, Vahedi M, Pourhoseingholi MA, Karkhane M, Kimiia Z, Pourhoseingholi A (2013). Direct medical care costs associated with patients diagnosed with chronic HCV. Hepat Mon.

[15] (2016-2017). Trading Economic Website.

[16] Long KH, Rubio-Tapia A, Wagie AE, Melton LJ 3rd, Lahr BD, Van Dyke CT (2010). The economics of coeliac disease: a population-based study. Aliment Pharmacol Ther.

[17] Green PH, Cellier C (2007). Celiac disease. N Engl J Med.

[18] Ehsani-Ardakani MJ, Rostami Nejad M, Villanacci V, Volta U, Manenti S, Caio G (2013). Gastrointestinal and non-gastrointestinal presentation in patients with celiac disease. Arch Iran Med.

